# A Multi-Breed Genome-Wide Association Analysis for Canine Hypothyroidism Identifies a Shared Major Risk Locus on CFA12

**DOI:** 10.1371/journal.pone.0134720

**Published:** 2015-08-11

**Authors:** Matteo Bianchi, Stina Dahlgren, Jonathan Massey, Elisabeth Dietschi, Marcin Kierczak, Martine Lund-Ziener, Katarina Sundberg, Stein Istre Thoresen, Olle Kämpe, Göran Andersson, William E. R. Ollier, Åke Hedhammar, Tosso Leeb, Kerstin Lindblad-Toh, Lorna J. Kennedy, Frode Lingaas, Gerli Rosengren Pielberg

**Affiliations:** 1 Science for Life Laboratory, Department of Medical Biochemistry and Microbiology, Uppsala University, Uppsala, Sweden; 2 Department of Basic Sciences and Aquatic Medicine, Norwegian University of Life Sciences, Oslo, Norway; 3 Centre for Integrated Genomic Medical Research, The University of Manchester, Manchester Academic Health Science Centre, Manchester, United Kingdom; 4 Institute of Genetics, Vetsuisse Faculty, University of Bern, Bern, Switzerland; 5 Department of Animal Breeding and Genetics, Swedish University of Agricultural Sciences, Uppsala, Sweden; 6 Department of Medicine (Solna), Karolinska Institutet, Stockholm, Sweden; 7 Department of Clinical Sciences, Swedish University of Agricultural Sciences, Uppsala, Sweden; 8 Broad Institute of MIT and Harvard, Cambridge, Massachusetts, United States of America; The Ohio State University, UNITED STATES

## Abstract

Hypothyroidism is a complex clinical condition found in both humans and dogs, thought to be caused by a combination of genetic and environmental factors. In this study we present a multi-breed analysis of predisposing genetic risk factors for hypothyroidism in dogs using three high-risk breeds—the Gordon Setter, Hovawart and the Rhodesian Ridgeback. Using a genome-wide association approach and meta-analysis, we identified a major hypothyroidism risk locus shared by these breeds on chromosome 12 (p = 2.1x10^-11^). Further characterisation of the candidate region revealed a shared ~167 kb risk haplotype (4,915,018–5,081,823 bp), tagged by two SNPs in almost complete linkage disequilibrium. This breed-shared risk haplotype includes three genes (*LHFPL5*, *SRPK1* and *SLC26A8*) and does not extend to the dog leukocyte antigen (DLA) class II gene cluster located in the vicinity. These three genes have not been identified as candidate genes for hypothyroid disease previously, but have functions that could potentially contribute to the development of the disease. Our results implicate the potential involvement of novel genes and pathways for the development of canine hypothyroidism, raising new possibilities for screening, breeding programmes and treatments in dogs. This study may also contribute to our understanding of the genetic etiology of human hypothyroid disease, which is one of the most common endocrine disorders in humans.

## Introduction

The domestic dog occupies an important position as a companion animal for humans, however for research scientists it also provides a unique comparative resource for genetic studies of phenotypic variation and development of diseases comparable to those in humans [1–4]. The genomic structure of domestic dogs, formed through domestication and intense breed-creation, is highly amenable to the identification of causal genetic loci in the same way as studies performed using other domestic animals [5–7]. Dogs share environmental factors with humans to a greater extent than any other domestic species, making them particularly suitable for comparative studies of complex diseases. The canine genome has been sequenced [5] and subsequently molecular tools for large-scale genotyping and analysis have been developed [8, 9]; large datasets describing gene expression are also publicly available [10, 11].

One of the most frequent endocrine diseases affecting both humans and dogs is hypothyroidism, a disorder in which the thyroid gland fails to produce sufficient amounts of thyroid hormones [12, 13]. Symptoms in human as well as in dogs are generally non-specific, including tiredness, weight gain and poor ability to tolerate cold, reflecting the key function of thyroid hormones in regulating the metabolism of the body [14, 15]. A common cause of human hypothyroidism worldwide is insufficient levels of dietary iodine [16]. However, the autoimmune disease called Hashimoto’s thyroiditis (HT) is also a common cause of hypothyroidism, especially in countries with sufficient daily intake of iodine [17]. The equivalent of HT in dogs is called canine lymphocytic thyroiditis (CLT). CLT is characterised by infiltration of B and T lymphocytes into the thyroid gland and the presence of circulating autoantibodies against thyroglobulin (TgAA), resulting in the progressive destruction of the thyroid [18–21]. Hypothyroidism in dogs may also be caused by thyroid idiopathic atrophy, characterised by a degenerative rather than autoimmune process, thought to potentially represent an end-stage of CLT [22]. The observation that some purebred dog breeds (primarily medium to large size breeds) are more predisposed to develop hypothyroidism suggests an underlying genetic risk for developing the disease. Breeds with high risk to develop hypothyroidism include the English Setter, Rhodesian Ridgeback, Giant Schnauzer, Hovawart, Old English Sheepdog, Boxer, Doberman Pinscher, Gordon Setter and the Beagle [18, 23–27]. The disease also shows notable familial clustering, further supporting the presence of a hereditary component [28]. Identifying the underlying genetic etiology of hypothyroidism in both humans and dogs is of major interest. Associations between major histocompatibility complex class II gene polymorphisms and hypothyroidism have previously been revealed in both humans (HLA) [29, 30] and dogs (DLA) [31, 32]. However, the reported DLA allele associations with CLT do not fully explain the susceptibility seen in all dog breeds. Furthermore, the contribution of DLA risk alleles to the development of hypothyroidism in specific breeds only explains a proportion of the underlying genetic risk for CLT. This indicates the presence of additional genetic risk factors and thus suggests a complex nature for the disease [22]. Congenital hypothyroidism in humans is associated with variants in genes including *DUOX2*, *PAX8*, *SLC5A5*, *TG*, *TPO*, *TSHB* and *TSHR* [33]. Genetic variants in the *TPO* gene are also associated with this rare type of hypothyroidism in Toy Fox and Tenterfield Terriers [34, 35].

Here we present a primary genome-wide association (GWA) study of hypothyroidism in three high-risk dog breeds, designed to identify novel susceptibility genes of the disease. This is the first time a major risk locus contributing to a complex disease has been identified across several dog breeds and confirmed as shared by using a meta-analysis approach.

## Materials and Methods

### Collection of samples and DNA preparation

EDTA blood and serum samples were collected from three hypothyroidism high-risk breeds. These breeds were Gordon Setter (GS) from Norway, Hovawart (HV) from several European countries, and Rhodesian Ridgeback (RR) from the United States of America (S1 Table). Preliminary characterisation of RR cohort is presented by Massey J.P. [36].

Samples were collected at veterinary clinics after obtaining owner’s written consent. Sampling routines appropriate for each of the different countries were followed (Norway: FOR-2010-08-06-1147; Sweden: Swedish Animal Ethical Committee No. C139/9 and C2/12 and Swedish Animal Welfare Agency No. 31-4714/09 and 31-998/12; Switzerland: Canton of Bern No. 23/10; United Kingdom and United States of America: ethical permit not needed for leftover samples originally collected for veterinarian purposes). Genomic DNA was extracted from the EDTA blood samples using QIAamp DNA Blood Mini/Midi extraction kit, QiaSymphony DNA midikit (both from Qiagen, Hilden, Germany) or E.Z.N.A blood DNA kit (Omega Bio-Tek, Norcross, GA, USA) following the manufacturers’ recommendations, and subsequently stored at -20°C. Serum was separated from clotted blood by centrifugation and stored at -20°C.

### Phenotyping

Dogs were initially classified as cases or controls based on clinical diagnoses from expert veterinarians certified in veterinary internal medicine. The clinical diagnoses of cases were subsequently supported by thyroid typical serological measurements (thyroid-stimulating hormone (TSH) and free thyroxine (fT4)) using Siemens IMMULITE Immunoassay System [31, 37]. In order to be classified as cases, dogs had to present with increased levels of TSH (> 40 mU/L) and reduced levels of fT4 (< 7 pmol/L), whereas controls had to be older than seven years of age. A subsequent careful survey of clinical records and/or questionnaires completed by dog owners was performed to exclude cases with another condition potentially influencing the dog’s wellbeing as well as controls with a history of immune-mediated conditions.

### SNP genotyping and quality control

The initial genetic datasets related to 165 GS, 74 HV and 92 RR individuals (S1 Table), and were genotyped using the Illumina 170 k CanineHD BeadChip (Illumina, San Diego, CA, USA) at the same technology platform. All SNP-positions are given according to the dog CanFam3.1 assembly [11].

Genotyping data quality control (QC) was carried out for each breed separately, using R v3.0.2 [38] and GenABEL v1.8–0 [39]. Firstly, an individual-based QC step was performed to identify potential duplicated samples and samples with gender discrepancies. Secondly, a marker-based QC was performed, including: pruning of the total set of SNPs according to minor allele frequency thresholds (MAF) (0.05 for all breeds), SNP and individual call rates (95% for all breeds), p-values (1x10^-5^ in GS, 1x10^-8^ in HV, 1x10^-3^ in RR) and false discovery rates for Hardy-Weinberg equilibrium (0.2 in all breeds). Moreover, each breed dataset was also checked for correlation between disease status and gender distribution. Fisher’s exact test and a phi coefficient were used to evaluate statistical significance and magnitude of correlation between such dichotomous variables (*i*.*e*. disease status and gender) [40, 41].

### Genome-wide association (GWA) analysis

A GWA analysis was performed on the quality controlled SNP datasets for GS, HV and RR breeds separately. All analytical steps were carried out using R v3.0.2 [38] and GenABEL v1.8–0 [39]. Using 2,000 randomly selected autosomal markers a genomic kinship matrix weighted by allele frequencies was computed in every breed. In all the breeds, we applied a standard linear mixed model, which was fitted using the polygenic_hglm function from the hglm package ver 2.0–8 [42], including the genomic kinship matrix as random effect. The mixed model approach is able to deal with both population structure and relatedness [43]. Breed-specific genomic kinship matrices were also used to project genetic distance between individuals into a plane using multidimensional scaling (MDS) and for subsequent plotting. For HV population, where samples had different geographic origins, we wanted to test whether this could have introduced any structure into the population. For this purpose, we followed an approach suggested by Tengvall and colleagues [3]. Shortly, we used K-means clustering to assign individuals to a predefined number of subpopulations. The number of clusters K = 2 (here subpopulations) was determined using a so-called scree test on a within-cluster sum of squares in a function of K (for details see [3]). Next, we used a linear mixed model with population as a fixed effect and genomic kinship as random effect. The statistical significance thresholds were evaluated as follows: (a) empirical genome-wide significance levels (P_genome_) obtained after 1000 permutations of the mixed model residuals (residualY returned by polygenic function in GenABEL) and (b) 95% empirical SNP distributions confidence intervals (CI_95_) as proposed by Karlsson and colleagues [1]. By permuting mixed model residuals, we maintained the connection between phenotypes and fixed effects [44], thus being able to evaluate the significance of only the genetic effects. For each single-breed GWA study, a quantile-quantile (QQ) plot was produced in R v3.0.2 and a Manhattan plot was generated using the R package qqman [45]. The independence of the signal was verified by association analysis conditioned on the genotype of the most significantly associated SNP (top SNP) for each breed separately.

Breed-specific associated loci were defined based on pairwise linkage disequilibrium (LD) estimates (R^2^ ≥ 0.7) of the three breed-specific top SNPs to SNPs in CFA12.

### Meta-analysis of genome-wide association

GWA meta-analysis of the three independent datasets (breeds) was carried out using MetABEL v0.2.0 [39], a part of the R statistical suite v3.0.2 [38]. Assuming the associated shared allelic effect being the same in each dataset, MetABEL performs a fixed effects meta-analysis, where each study is weighted according to the inverse of its’ squared standard error in order to maximise the power of discovery [46]. We created an MDS plot, displaying the samples belonging to the three different breeds as subpopulations, a QQ plot, showing the degree of deviation of the associated SNPs compared to their null distribution, and a Manhattan plot, showing the genome-wide association signals, as described above.

### Haplotype analysis

The minimal risk haplotype, shared across breeds, was identified in the associated locus from the meta-analysis. Firstly, genotypes of the shared associated region were imputed (if missing) and phased into haplotypes in each breed separately using fastPHASE [47]. At this stage the phenotype of each individual was used as a covariate, in order to avoid prediction of spurious haplotypes. Thereafter, the risk haplotypes present in cases and non-risk haplotypes present in controls were identified based on the genotype at the meta-analysis top SNP. Starting from the meta-analysis top SNP and walking both up- and downstream, we then identified the SNP-positions where the risk haplotype was broken by a recombination event (*i*.*e*. two alternative alleles were present on both risk and non-risk haplotypes). This was done separately for each breed, and thereafter the minimal shared risk haplotype across breeds defined.

Two SNPs tagging the associated risk haplotype across breeds were analysed for association with the phenotype as both haplotypes and genotypes using Pearson’s Chi-squared and Fisher’s exact tests respectively [40, 48].

## Results

### GWA study sample cohort

We performed genotyping of 165 GS, 74 HV and 92 RR individuals for ~170,000 SNP markers distributed across the entire canine genome. The individual-based QC indicated 1 HV and 18 GS samples due to gender discrepancies between phenotypic and genetic data, retaining 147 GS (63 cases and 84 controls), 73 HV (44 cases and 29 controls) and 92 RR (38 cases and 54 controls) individuals for further analysis (S1 Table).

The marker-based QC removed the majority of SNPs due to MAF < 0.05, whereas SNPs were also removed due to call rate < 95% and deviation from the Hardy-Weinberg equilibrium (S2 Table). The final datasets consisted of 110,221 markers in GS, 100,864 in HV and 103,612 in RR cohorts.

We did not identify a significant correlation between phenotype and gender distribution in any of the breeds (p = 0.9, phi coefficient = 0.02 in GS; p = 0.1, phi coefficient = 0.19 in HV and p = 0.39, phi coefficient = 0.11 in RR).

### Single-breed GWA studies show association to CFA12 in all breeds

**Fig 1:**
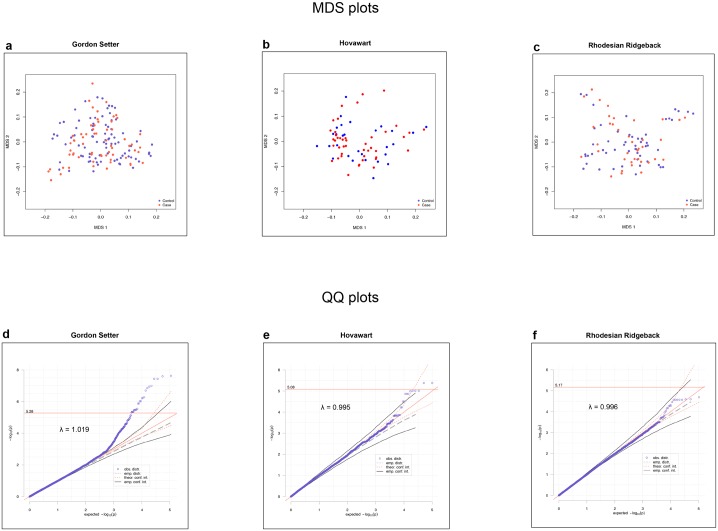
(a-c) MDS plots showing homogenous sample sets without presence of clustered samples in the (a) Gordon Setter (GS), (b) Hovawart (HV) and (c) Rhodesian Ridgeback (RR) breed cohorts. (d-f) QQ plots showing no inflation after the mixed model approach, and the empirical genome-wide significance thresholds (indicated by red lines and their corresponding values) and empirical 95% confidence intervals (CI_95_) in (d) GS λ = 1.019, (e) HV λ = 0.995 and (f) RR λ = 0.996.

Association analysis for hypothyroidism was performed in the three breeds separately. Population stratification was not detected in any of the breeds, as confirmed by the even distribution of cases and controls on the MDS plots (Fig 1a–1c). After the application of the mixed model approach, to account for population structure and cryptic relatedness common in dog breeds, no genetic inflation was observed in any of the breed-specific association tests (λ = 1.019 in GS, λ = 0.995 in HV and λ = 0.996 in RR) as presented on the QQ plots (Fig 1d–1f). The QQ plots also indicate the breed-specific statistical significance levels (see materials and methods).

**Fig 2:**
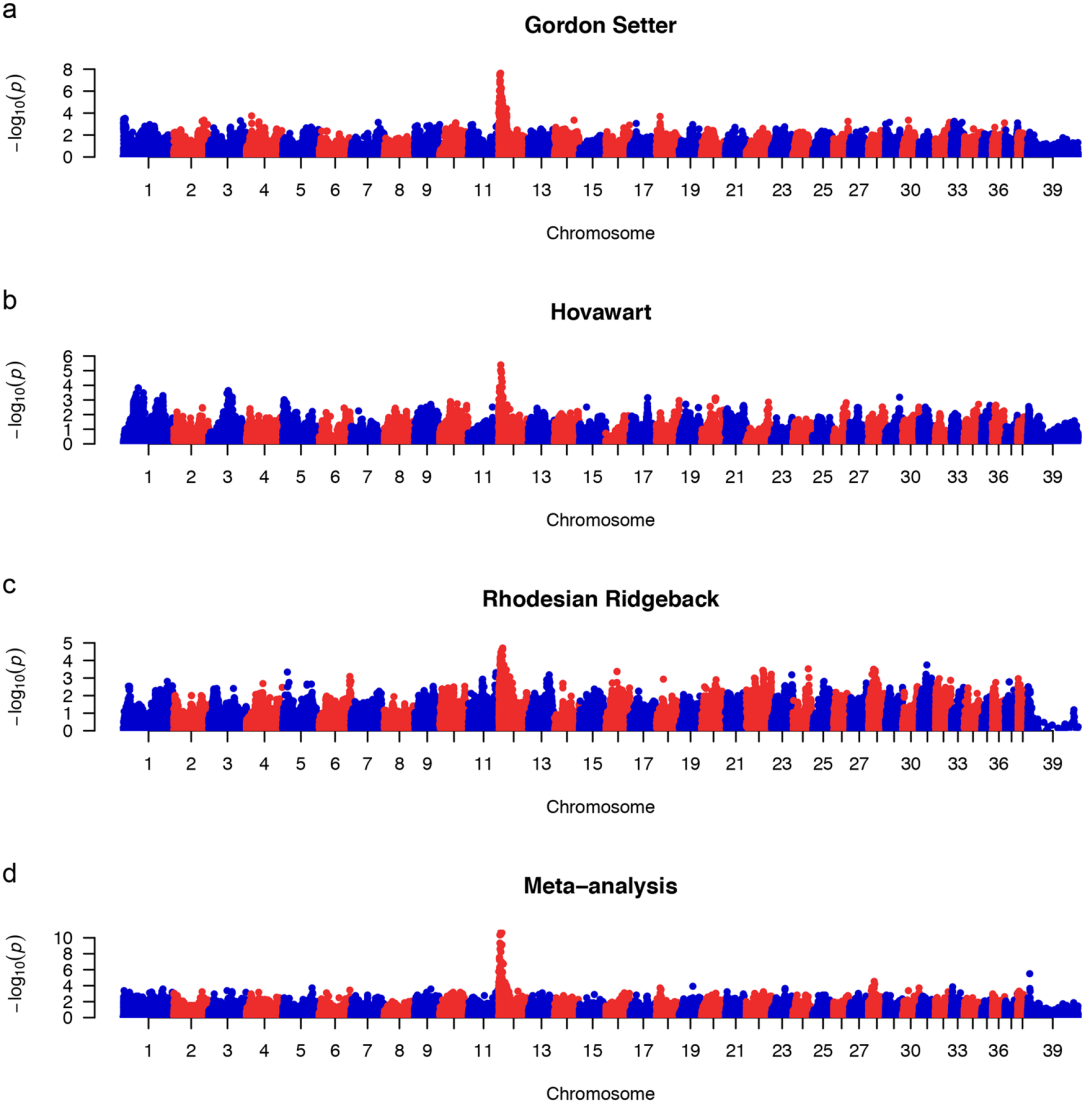
Manhattan plots showing a common peak on CFA12 in (a) GS p_raw_ = 2.4x10^-8^, (b) HV p_raw_ = 4.2x10^-6^, (c) RR p_raw_ = 2.0x10^-5^ and (d) multi-breed meta-analysis p_raw_ = 2.1x10^-11^.

We detected a significant genetic association in the GS and HV and a suggestive association in the RR to a region on CFA12 (Figs 1 and 2a–2c) with the following breed-specific top SNPs: GS 4,456,564 bp (p_raw_ = 2.4x10^-8^), HV 5,158,474 bp (p_raw_ = 4.2x10^-6^) and RR 9,336,752 bp (p_raw_ = 2.0x10^-5^) (Table 1). We found the most significant association in the GS breed cohort, reaching a Bonferroni corrected alpha = 5% significance level (p < 4.5x10^-7^), which is in any case considered excessively stringent in dog GWA studies. In the HV breed the association significance exceeded both empirical genome-wide and empirical CI_95_ levels, whereas in the RR breed the association was suggestive towards the CI_95_ empirical cut-off.

**Table 1:** Summary of the three single-breed GWA analysis results.

	GWA top SNP (bp)	P_raw_	OR	CI_OR_	P_genome_	CI_95_
**Gordon Setter**	4,456,564	2.4x10^-8^	4.0	2.1–7.0	< 5.2x10^-6^	< 2.0x10^-3^
**Hovawart**	5,158,474	4.2x10^-6^	5.0	2.1–13.1	< 8.3x10^-6^	< 3.2x10^-5^
**Rhodesian Ridgeback**	9,336,752	2.0x10^-5^	3.4	1.8–6.7	< 6.8x10^-6^	NA

P_raw_—uncorrected p-value for the strongest association, OR—Odds ratio, CI_OR_—OR 95% Confidence Intervals, P_genome_—empirical genome-wide significance levels obtained after 1000 permutations, CI_95_—95% empirical SNP distributions confidence intervals, NA—not applicable.

The GS and HV breed-specific top SNPs were present in the GWA analysis of all breeds, whereas the RR breed-specific top SNP was removed from GS and HV populations in marker-based QC process due to MAF < 0.05 (in GS population: MAF = 0.030, MAF_Cases_ = 0.047, MAF_Controls_ = 0.018; in HV population: MAF = 0.027, MAF_Cases_ = 0.034, MAF_Controls_ = 0.017) (S3 Table).

### Breed-specific associated regions overlap

**Fig 3:**
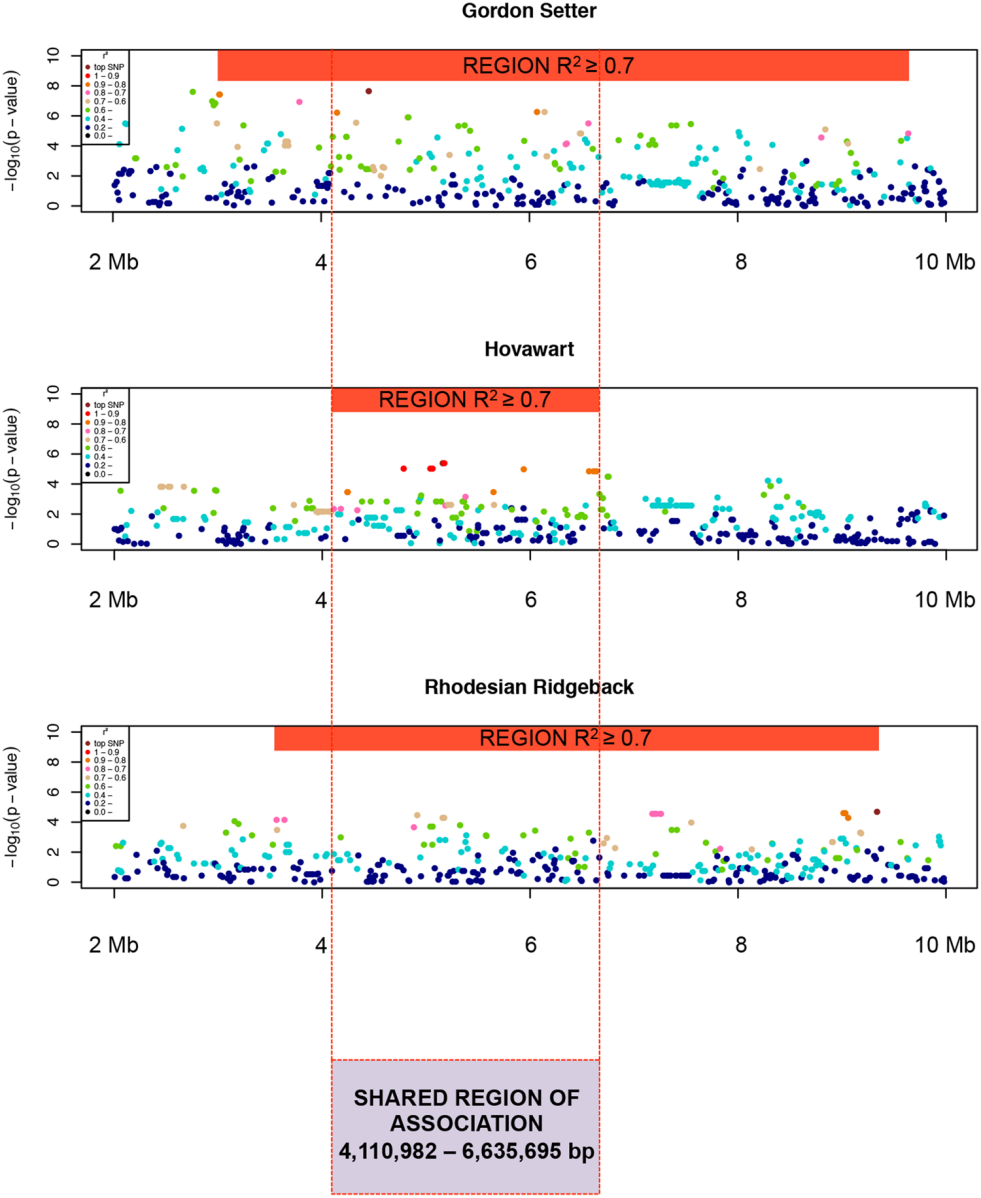
Breed-specific LD Manhattan plots of relevant region on CFA12, indicating R^2^-values of each SNP in respect to the highest associated markers. Orange bars are highlighting the associated regions (R^2^ ≥ 0.7) in GS (3,022,017–9,637,877 bp), HV (4,110,982–6,635,695 bp) and RR (3,561,421–9,336,752 bp). Dashed lines indicate the shared region of association (4,110,982–6,635,695 bp).

To define the location of associated regions on CFA12 in each of the three dog breeds, we performed an LD-analysis based on R^2^-values. By including the breed-specific SNP sets used for association analysis we determined the R^2^-value of each SNP on CFA12 to the respective breed-specific top SNP. Using a cutoff of R^2^ ≥ 0.7 as a signal of LD we defined the associated regions on CFA12 as following: GS 3,022,017–9,637,877 bp, HV 4,110,982–6,635,695 bp and RR 3,561,421–9,336,752 bp (Fig 3). The long associated regions in each breed (~2.5–6.6 Mb) were confirmed as single signals by conditional association analysis (S1 Fig). The shared associated region was defined as 4,110,982–6,635,695 bp (~2.5 Mb), corresponding to the associated region defined for the HV sample cohort (Fig 3).

### Meta-analysis confirms one major risk locus shared in all three breeds

**Fig 4:**
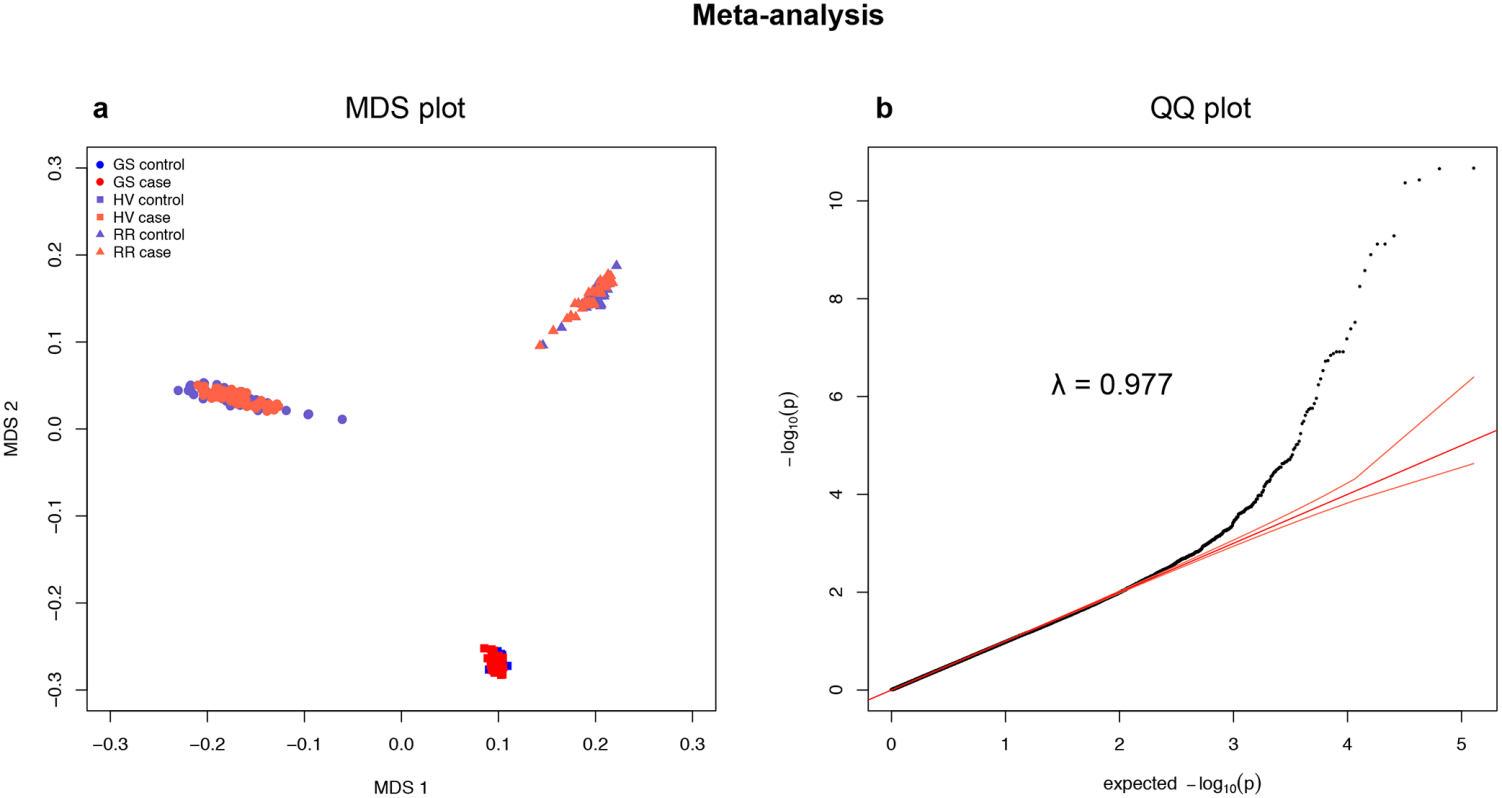
Meta-analysis (a) MDS plot showing genetic distances between GS, HV and RR sample sets and (b) QQ plot showing no inflation (λ = 0.977). The red lines show theoretical distribution in absence of association and theoretical confidence intervals.

Meta-analysis was performed across breeds (MDS plot Fig 4a), in order to identify the shared associated region and the top SNP. No genetic inflation was observed (λ = 0.977, QQ plot Fig 4b) and the most significant association (P_raw_ = 2.1x10^-11^) was detected at CFA12: 5,039,806 bp, located in the shared associated region identified above from the breed-specific GWA analyses (Fig 2d). Therefore, CFA12: 4,110,982–6,635,695 bp represents a candidate region for increased susceptibility to canine hypothyroidism in the GS, HV and the RR breeds. This region contains many genes (n > 40), which include candidate genes involved in cell survival, apoptosis and immunity (*e*.*g*. *DEF6*, *MAPK14*, *STK38* and *CDKN1A*).

### Haplotype analysis across breeds identifies a shared risk haplotype

**Fig 5:**
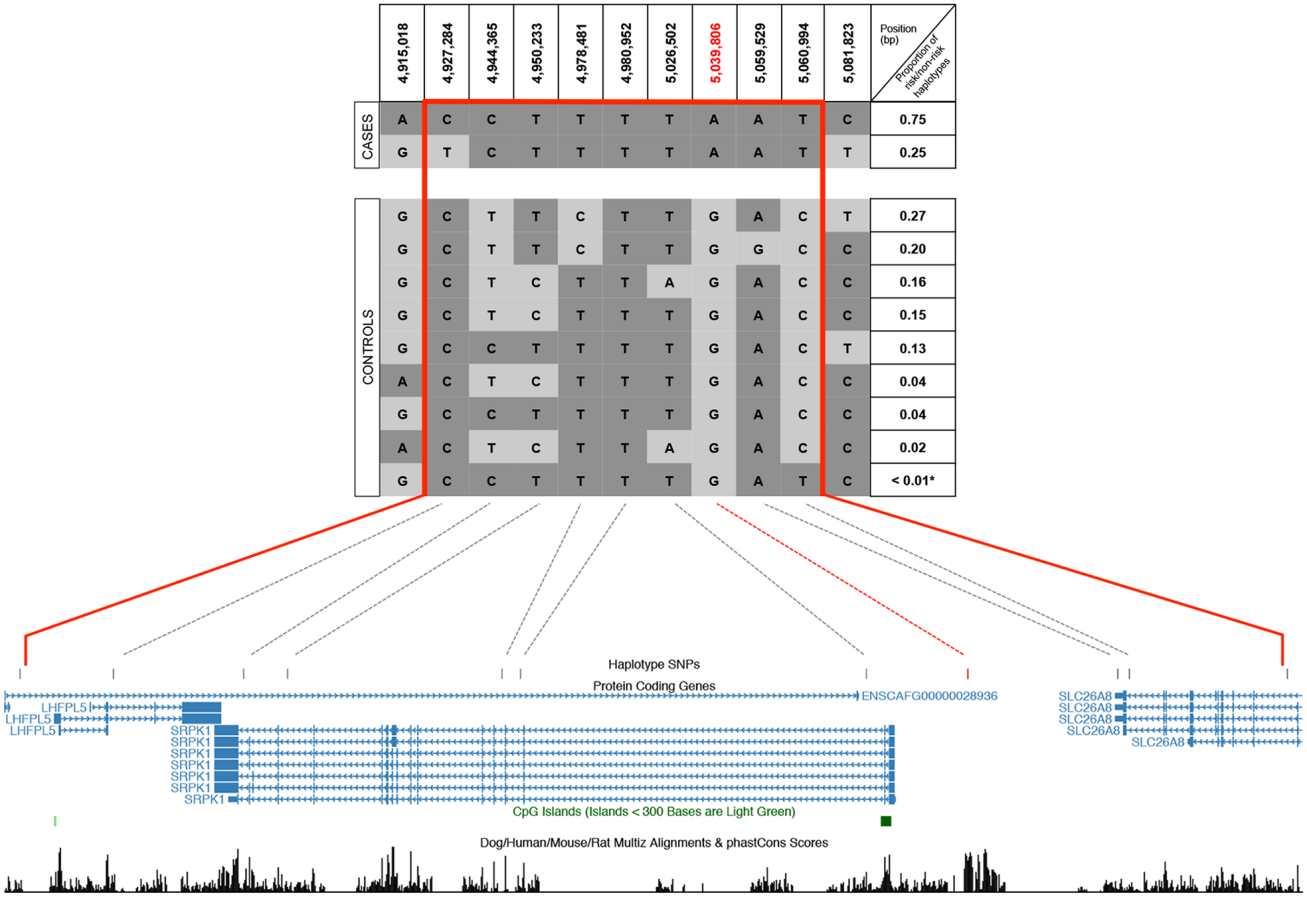
Haplotype analysis from all breeds identified two risk haplotypes in cases and nine non-risk haplotypes in controls. The upper panel indicates the alternative alleles in different grey shades, and the defined ~167 kb risk haplotype (4,915,018–5,081,823 bp) with red boundaries. Proportion of risk/non-risk haplotypes indicates the relative proportions of corresponding haplotypes among risk haplotypes determined in cases or non-risk haplotypes determined in controls, respectively. Star indicates a rare haplotype represented by only one chromosome. The lower panel shows the genomic location of the SNPs defining the risk haplotype in relation to protein coding genes (for each gene all the transcripts isoforms are included), CpG islands and conservation scores (figure adapted from: http://genome.ucsc.edu)

Haplotype analysis was performed in each breed separately using the meta-analysis top SNP as a tagging marker for risk and control haplotypes. We identified risk haplotypes for each breed as following: GS 4,915,018–5,081,823 bp, HV 4,906,914–5,081,823 bp and RR 3,496,085–5,158,474 bp; thereby defining the risk haplotype shared across breeds as 4,915,018–5,081,823 bp. The defined risk haplotype spans ~167 kb and harbours 9 SNPs. Fig 5 shows all risk haplotypes identified in cases and all non-risk haplotypes identified in controls from all three breeds. The ~167 kb haplotype harbours three genes, *LHFPL5*, *SRPK1* and *SLC26A8*, which have not previously been implicated, and are thereby novel, with respect to the development of hypothyroidism.

The meta-analysis top SNP (5,039,806 bp, G/A, located between *SRPK1* and *SLC26A8* genes) and another SNP in the vicinity (5,060,994 bp, C/T, located in the last intron of *SLC26A8*) showed a complete LD in risk haplotypes and nearly complete LD in control haplotypes across all three breeds (recombination indicated on only one chromosome, see Fig 5). We used these two SNPs and the phenotypes for the following association tests: haplotype-based (non-risk = GC, risk = AT) and genotype-based (homozygous non-risk = G/G and C/C, heterozygous = G/A and C/T, and homozygous risk = A/A and T/T). We identified a significant enrichment of the risk (AT) haplotype in cases versus controls both in each breed separately and across breeds (see Table 2 for p-values). Similarly, a significant association was also observed when associating genotypes of these SNPs to the phenotypic classes in each breed separately and across breeds (see Table 2 for p-values).

**Table 2:** Haplotype and genotype association to phenotypic classes.

		Haplotype				Genotype (5,039,806)*				Genotype (5,060,994)*			
		GC	AT	p-value	OR	G/G	G/A	A/A	p-value	C/C	C/T	T/T	p-value
**Gordon Setter**	cases 63	98 (0.77)	28 (0.23)	7.3x10^-4^	3.4 (CI = 1.6–7.5)	32 (0.52)	29 (0.48)	0 (0)	2.3x10^-5^	34 (0.54)	29 (0.46)	0 (0)	3.3x10^-5^
	controls84	154^#^ (0.92)	13 (0.08)			68 (0.85)	11 (0.14)	1 (0.01)		71 (0.85)	12 (0.14)	1 (0.01)	
**Hovawart**	cases 44	48 (0.55)	40 0.45)	5.3x10^-5^	6.0 (CI = 2.4–17.4)	10 (0.22)	28 (0.64)	6 (0.14)	1.0x10^-5^	10 (0.22)	28 (0.64)	6 (0.14)	1.0x10^-5^
	controls29	51 (0.88)	7 (0.12)			22 (0.77)	7 (0.23)	0 (0)		22 (0.77)	7 (0.23)	0 (0)	
**Rhodesian Ridgeback**	cases 38	43 (0.57)	33 (0.43)	8.0x10^-4^	3.2 (CI = 1.6–6.5)	13 (0.34)	17 (0.45)	8 (0.21)	3.0x10^-3^	13 (0.34)	17 (0.45)	8 (0.21)	3.0x10^-3^
	controls54	87 (0.81)	21 (0.19)			35 (0.65)	17 (0.31)	2 (0.04)		35 (0.65)	17 (0.31)	2 (0.04)	
**Combined breeds**	cases 145	189 (0.65)	101 (0.35)	4.5x10^-11^	3.8 (CI = 2.5–5.9)	55 (0.38)	74 (0.52)	14 (0.1)	1.5x10^-11^	57 (0.39)	74 (0.51)	14 (0.1)	3.0x10^-11^
	controls167	292 (0.88)	41 (0.12)			125 (0.77)	35 (0.21)	3 (0.02)		128 (0.77)	36 (0.21)	3 (0.02)	

Association analysis of two tagging SNPs as haplotypes and genotypes to phenotypic classes in breeds separately and combined. Numbers in columns of haplotype indicate the number of chromosomes, whereas numbers in columns of genotypes indicate the number of individuals. Numbers in brackets show proportion of chromosomes/genotypes in cases and controls. OR—odds ratios, CI—confidence intervals.

* Differences in the number of genotypes for SNP 5,039,806 and 5,060,994 are due to missing genotypes

^#^ One missing chromosome due to a recombination event (*i*.*e*. AC-haplotype)

## Discussion

In this study we present the first GWA analysis identifying a major shared risk locus for the development of canine hypothyroidism in three high-risk dog breeds. By adapting a multi-breed approach, we have identified a shared risk haplotype for a complex disease. The possibility of using such an approach was proposed in 2007, when Karlsson and colleagues [8] used a monogenic white coat colour as an example trait. The authors used a one-breed GWA study, followed by an approach to narrow down the candidate region by including another breed with the same phenotype. Since then, studies have used an inter-breed approach to map canine brachycephaly [49], and a multi-breed common pathway analysis to identify genes behind canine osteosarcoma [1]. The integrated GWA and meta-analysis approach presented here is to our knowledge the first successful study identifying a risk locus involved in the development of a complex disease across dog breeds. Such meta-analysis has been widely used in human genetics, in which the detected loci explain only a small proportion of the genetic contribution to the respective complex traits [46, 50].

In our study, the meta-analysis validated and corroborated the single-breed GWA analysis results, as well as identified a ~167 kb risk haplotype for canine hypothyroidism shared among three high-risk breeds. Although the identified risk haplotype explains a large proportion of the cases in our study cohorts, there are likely additional risk factors contributing to the development of hypothyroidism in dogs and even in the breeds included in our study. The risk haplotype is located on CFA12, starting approximately 2 Mb downstream of the DLA gene cluster. DLA haplotypes on CFA12 in dogs [31, 32] and HLA haplotypes on HSA6 in humans [51–53] have already been associated with an increased risk of developing hypothyroidism in both species. Wilbe and colleagues [32] investigated the role of DLA haplotypes in hypothyroid Hovawart dogs without identifying any association and observing low genetic variation in the region. Such association analyses are considered difficult due to complex LD structure of the region [54], and also because of potentially spurious association signals obtained when the number of haplotypes in the region is limited [55]. Our study provides strong evidence of a canine hypothyroidism risk locus on a region of CFA12 not harbouring the DLA region.

We identified a ~167 kb risk haplotype associated with hypothyroidism in all the three breeds included in our study. Thus, we have determined a haplotype carrying the putative causative mutation(s), which could be located either in coding or regulatory region(s). The haplotype contains three genes (*LHFPL5*, *SRPK1* and *SLC26A8*) with functions that are not explicitly obvious in the development of hypothyroidism. However, they could pave the way to the characterisation of entirely novel pathways and mechanisms having a role in the etiology of this disease. *LHFPL5* is a gene encoding for lipoma HMGIC fusion partner-like 5. Mutations in this gene result in deafness in humans [56, 57], and in mice [58]. *SRPK1* (SRSF protein kinase 1) encodes a serine/arginine protein kinase specific for the SR (serine/arginine-rich domain) family of splicing factors. It has been shown as being an important factor in tumorigenesis [59], viral infection [60, 61] and apoptosis [62]. *SLC26A8* (solute carrier family 26 member 8) is a member of the *SLC26* gene family of anion transporters, which are well-conserved in both gene structure and protein length. Variants in one member of this gene family, specifically in *SLC26A4*, have been shown to cause a genetic disorder called Pendred syndrome, characterised by goitre and occasionally also hypothyroidism [63]. Variants in *SLC26A8* have been implicated to cause asthenozoospermia (reduced sperm motility) via altered interaction with CFTR (cystic fibrosis transmembrane conductance regulator) [64]. Variants in the *CFTR* gene are known to cause cystic fibrosis, often comorbid with iodine deficiency and subclinical hypothyroidism [65, 66], thereby indicating a potential functional link to our phenotype of interest. Even though the expression of *SLC26A8* gene is reported to be restricted to spermatocytes [67], several publicly available databases (the Human Protein Atlas (www.proteinatlas.org) [68], BioGPS database (www.biogps.org) [69] suggest expression in a wide range of tissues and cell-lines. Consequently, *SLC26A8* emerges as the strongest candidate gene within the associated risk haplotype.

The associated haplotype contains many conserved elements based on analysis of 29 different mammalian species [70] and some GC-rich regions potentially functioning as CpG islands. The corresponding human region indicates abundant chromatin functional structures and transcription factor binding sites. It is possible that, despite being located in the ~167 kb haplotype identified in our study, the putative causative mutation(s) for canine hypothyroidism may be regulating the expression of a gene which may lie outside the borders of the haplotype.

On average we would expect haplotypes within dog breeds being around 1 Mb, and across several dog breeds about 10–100 kb [5]. However the breed-specific associated regions and the associated haplotype identified in our study are longer. One of the reasons for these long haplotypes may be selection *i*.*e*. favorable genetic information in the region keeping the haplotypes intact. The three genes located in the ~167 kb risk haplotype are all involved in basic physiological processes, suggesting that any of them might have represented a favourable target for selection. Therefore, it is possible that the detrimental hypothyroidism risk factor may have been “hitchhiking” together with the selected locus during dog domestication or breed-creation. In a study designed to identify domestication selection signals, the authors reported no sweep signals between wolves and dogs in the ~167 kb hypothyroidism risk haplotype implicated in our study [71]. Indeed, the hypothyroidism risk alleles are absent from the wolf population used in the Axelsson study, thereby not supporting the possible prior domestication origin of these alleles (http://genome.ucsc.edu, public track hub: Broad Improved Canine Annotation v1, track: Axelsson SNPs) [11, 71]. Additionally, hypothyroidism risk alleles have been reported in dog breeds with low risk for developing hypothyroidism [9]. Therefore we propose that the putative canine hypothyroidism risk factor appeared after domestication and before breed-creation, since gene flow between breeds included in our study is very unlikely. Further research on the evolutionary and demographic history of the hypothyroidism risk factor in different dog breeds is of utmost interest and should help us to strengthen this hypothesis.

In summary, we have demonstrated the notable potential of the integrated GWA and meta-analysis approach for detecting genetic loci underlying complex diseases in dogs. Further characterisation of the risk haplotype for canine hypothyroidism present in the Gordon Setter, Hovawart and the Rhodesian Ridgeback populations used in this study is necessary in order to extensively and deeply characterise the locus from a genomic and a functional point of view. We expect that future work focusing on this genomic region may identify a shared functional variant(s) increasing the risk of developing hypothyroidism in dogs. The identification of the functional variant(s) may contribute to the wellbeing not only of dogs, via breeding strategies, but also benefit human research, via identification of new potential genetic risk factors, pathways and treatment strategies for hypothyroidism.

## Supporting Information

S1 Fig QQ and Manhattan plots after conditioning breed-specific association analyses for respective top SNP. (TIF)

S1 Table Overview of samples used in the study. (DOCX)

S2 Table SNP-based quality control summary. (DOCX)

S3 Table Associations of breed-specific top SNP genotypes to phenotypic classes in the different breeds. (DOCX)
