# Supplementary figures and images for: A Multi-Breed Genome-Wide Association Analysis for Canine Hypothyroidism Identifies a Shared Major Risk Locus on CFA12
Source: PLoS One. 2015 Aug 11;10(8):e0134720. doi: 10.1371/journal.pone.0134720 (PMC4532498; doi:10.1371/journal.pone.0134720)

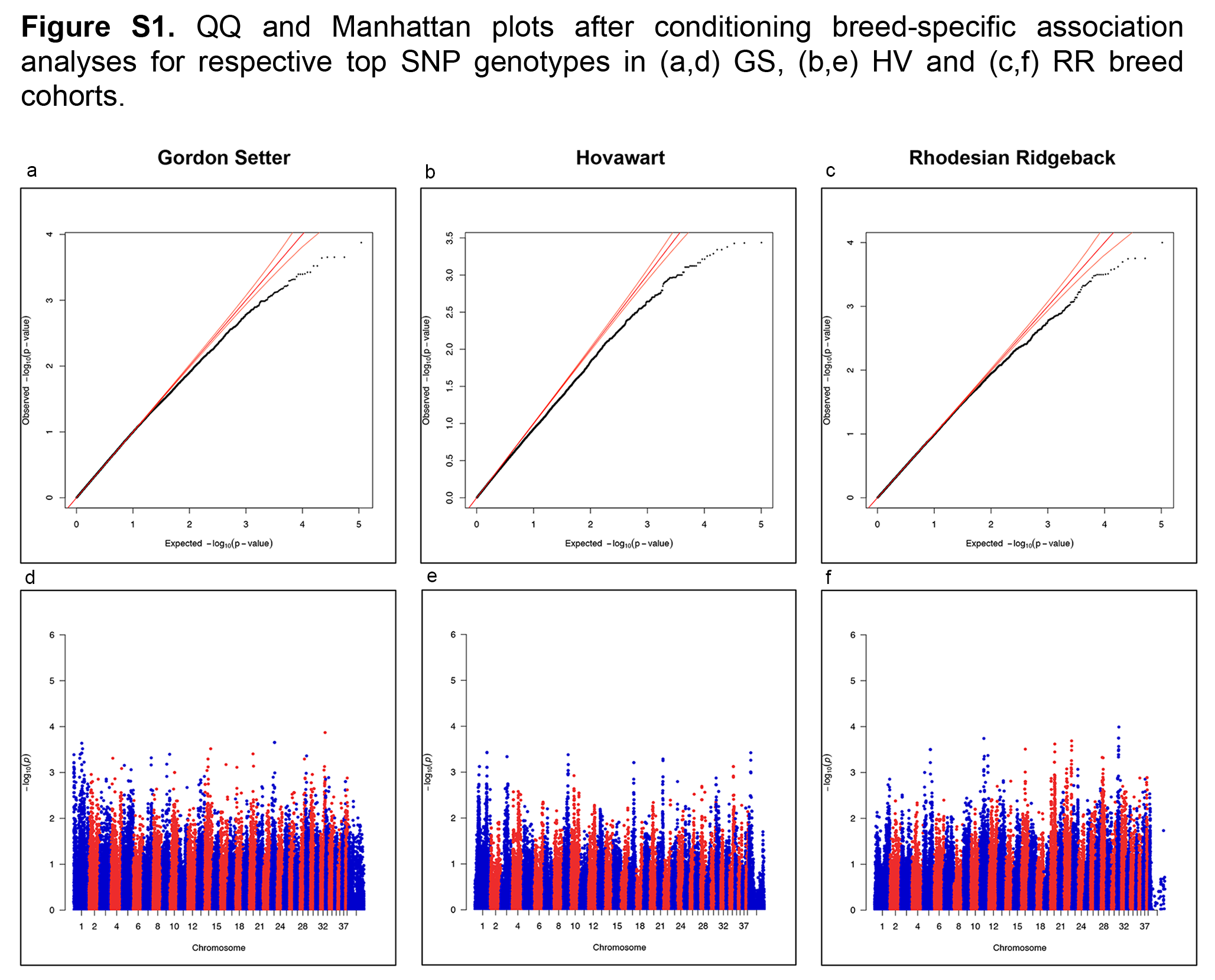

Supplement: S1 Fig — (TIF) [file pone.0134720.s001.tif]
