# Supplementary material for: A Multi-Breed Genome-Wide Association Analysis for Canine Hypothyroidism Identifies a Shared Major Risk Locus on CFA12
Source: PLoS One. 2015 Aug 11;10(8):e0134720. doi: 10.1371/journal.pone.0134720 (PMC4532498; doi:10.1371/journal.pone.0134720)
Supplement: S1 Table — (DOCX) [file pone.0134720.s002.docx]

**Table S1.** Overview of samples used in the study. Numbers indicate the numbers of case and control individuals before and after quality control (QC).

| Breed | Country of origin | Phenotype | Initial nr. of dogs | Nr. of dogs after QC |
| --- | --- | --- | --- | --- |
| Gordon Setter | Norway | Case | 73 | 63 |
|  |  | Control | 92 | 84 |
| Hovawart | Sweden | Case | 22 | 22 |
|  |  | Control | 18 | 18 |
|  | Germany | Case | 15 | 14 |
|  |  | Control | 10 | 10 |
|  | Switzerland | Case | 2 | 2 |
|  |  | Control | 1 | 1 |
|  | France | Case | 2 | 2 |
|  |  | Control | 0 | 0 |
|  | UK | Case | 2 | 2 |
|  |  | Control | 0 | 0 |
|  | Finland | Case | 1 | 1 |
|  |  | Control | 0 | 0 |
|  | Netherland | Case | 1 | 1 |
|  |  | Control | 0 | 0 |
| Rhodesian Ridgeback | USA | Case | 38 | 38 |
|  |  | Control | 54 | 54 |
