# Supplementary material for: A Multi-Breed Genome-Wide Association Analysis for Canine Hypothyroidism Identifies a Shared Major Risk Locus on CFA12
Source: PLoS One. 2015 Aug 11;10(8):e0134720. doi: 10.1371/journal.pone.0134720 (PMC4532498; doi:10.1371/journal.pone.0134720)
Supplement: S2 Table — (DOCX) [file pone.0134720.s003.docx]

**Table S2. Marker-based quality control summary.** Number of SNPs removed from analysis based on low minor allele frequency (NoMAF, <5%), low call rate (NoCall, <95%) and deviation from Hardy-Weinberg equilibrium (NoHWE).

|  |  | **NoCall** | **NoMAF** | **NoHWE** |
| --- | --- | --- | --- | --- |
| **Gordon Setter** | **NoCall** | 7,184 | 1,641 | 434 |
|  | **NoMAF** | NA | 53,280 | 23 |
|  | **NoHWE** | NA | NA | 91 |
| **Hovawart** | **NoCall** | 2,968 | 1,819 | 430 |
|  | **NoMAF** | NA | 67,439 | 0 |
|  | **NoHWE** | NA | NA | 41 |
| **Rhodesian Ridgeback** | **NoCall** | 2,308 | 462 | 381 |
|  | **NoMAF** | NA | 62,181 | 564 |
|  | **NoHWE** | NA | NA | 4,161 |
