# Supplementary material for: A Multi-Breed Genome-Wide Association Analysis for Canine Hypothyroidism Identifies a Shared Major Risk Locus on CFA12
Source: PLoS One. 2015 Aug 11;10(8):e0134720. doi: 10.1371/journal.pone.0134720 (PMC4532498; doi:10.1371/journal.pone.0134720)
Supplement: S3 Table — (DOCX) [file pone.0134720.s004.docx]

**Table S3.** Associations of breed-specific top SNP genotypes to phenotypic classes in the different breeds.

|  |  | **GS top SNP (4,456,564)*** | | | | **HV top SNP (5,158,474)*** | | | | **RR top SNP (9,336,752)*** | | | |
| --- | --- | --- | --- | --- | --- | --- | --- | --- | --- | --- | --- | --- | --- |
|  |  | **C/C** | **A/C** | **A/A** | **p-value** | **G/G** | **G/A** | **A/A** | **p-value** | **C/C** | **C/T** | **T/T** | **p-value** |
| **Gordon Setter** | cases 63 | 13 (0.21) | 39 (0.62) | 11 (0.17) | 2.4x10^-8^ | 14 (0.22) | 34 (0.54) | 15 (0.24) | 0.88 | 57 (0.90) | 6 (0.1) | 0 (0) | NA |
|  | controls84 | 56 (0.67) | 23 (0.27) | 5 (0.06) |  | 20 (0.24) | 40 (0.48) | 24 (0.28) |  | 80 (0.96) | 3 (0.04) | 0 (0) |  |
| **Hovawart** | cases 44 | 18 (0.41) | 21 (0.48) | 5 (0.11) | 1.8x10^-2^ | 7 (0.16) | 31 (0.70) | 6 (0.14) | 4.2x10^-6^ | 41 (0.93) | 3 (0.07) | 0 (0) | NA |
|  | controls29 | 7 (0.24) | 12 (0.41) | 10 (0.35) |  | 20 (0.69) | 9 (0.31) | 0 (0) |  | 28 (0.97) | 1 (0.03) | 0 (0) |  |
| **Rhodesian Ridgeback** | cases 38 | 12 (0.32) | 19 (0.51) | 6 (0.17) | 0.42 | 4 (0.11) | 18 (0.47) | 16 (0.42) | 5.1x10^-5^ | 7 (0.18) | 15 (0.39) | 16 (0.43) | 2.0x10^-5^ |
|  | controls54 | 15 (0.28) | 27 (0.5) | 12 (0.22) |  | 26 (0.48) | 23 (0.43) | 5 (0.09) |  | 25 (0.46) | 25 (0.46) | 4 (0.08) |  |

Association analysis of breed-specific GWA analysis top SNPs to phenotypic classes in the different breeds. Numbers in columns of genotypes indicate the number of individuals, whereas numbers in brackets show proportion of genotypes in cases and controls.

* Differences in the number of genotypes for different SNPs are due to missing genotypes

NA Not applicable due to MAF < 0.05
